# Mining literature for a comprehensive pathway analysis: A case study for retrieval of homocysteine related genes for genetic and epigenetic studies

**DOI:** 10.1186/1476-511X-5-1

**Published:** 2006-01-23

**Authors:** Priyanka Sharma, RD Senthilkumar, Vani Brahmachari, Elayanambi Sundaramoorthy, Anubha Mahajan, Amitabh Sharma, Shantanu Sengupta

**Affiliations:** 1 Department of Proteomics and Structural Biology, Institute of Genomics and Integrative Biology, Mall Road, Delhi-110007, India; 2 Dr. B. R. Ambedkar Centre for Biomedical Research, University of Delhi, Delhi-110007, India

## Abstract

Homocysteine is an independent risk factor for cardiovascular diseases. It is also known to be associated with a variety of complex disorders. While there are a large number of independent studies implicating homocysteine in isolated pathways, the mechanism of homocysteine induced adverse effects are not clear. Homocysteine-induced modulation of gene expression through alteration of methylation status or by hitherto unknown mechanisms is predicted to lead to several pathological conditions either directly or indirectly. In the present manuscript, using literature mining approach, we have identified the genes that are modulated directly or indirectly by an elevated level of homocysteine. These genes were then placed in appropriate pathways in an attempt to understand the molecular basis of homocysteine induced complex disorders and to provide a resource for selection of genes for polymorphism screening and analysis of mutations as well as epigenetic modifications in relation to hyperhomocysteinemia. We have identified 135 genes in 1137 abstracts that either modulate the levels of homocysteine or are modulated by elevated levels of homocysteine. Mapping the genes to their respective pathways revealed that an elevated level of homocysteine leads to the atherosclerosis either by directly affecting lipid metabolism and transport or via oxidative stress and/or Endoplasmic Reticulum (ER) stress. Elevated levels of homocysteine also decreases the bioavailability of nitric oxide and modulates the levels of other metabolites including S-adenosyl methionine and S-adenosyl homocysteine which may result in cardiovascular or neurological disorders. The ER stress emerges as the common pathway that relates to apoptosis, atherosclerosis and neurological disorders and is modulated by levels of homocysteine. The comprehensive network collated has lead to the identification of genes that are modulated by homocysteine indicating that homocysteine exerts its effect not only through modulating the substrate levels for various catalytic processes but also through regulation of expression of genes involved in complex diseases.

## Review

**Figure 1:**
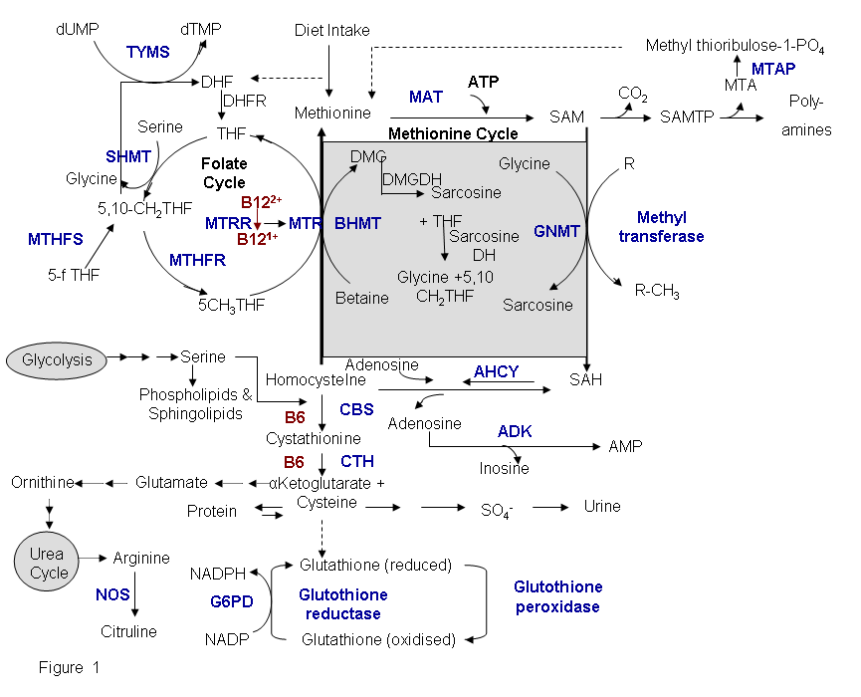
**Methionine-Homocysteine metabolism and related pathways**. A representation of the methionine cycle (central), transulfuration pathway and its connection to folate cycle, glycolysis pathway and urea cycle. The genes marked in blue have been identified by literature based searches as mentioned in the methods section. The solid and dotted lines indicate direct and indirect (multi step) interaction/ conversion respectively.

Elevated levels of homocysteine (hyperhomocysteinemia) has been implicated as an independent risk factor for cardiovascular disease [1,2] and is associated with various other diseases and/or clinical conditions including Alzheimer's disease [3], neural tube defects [4], schizophrenia [5], end-stage renal disease [6], osteoporosis [7] and non-insulin-dependent diabetes [8,9]. Homocysteine, a thiol containing amino acid, is formed during methionine metabolism in the cell. It is a key branch-point intermediate in the ubiquitous methionine cycle, the function of which is to generate one-carbon methyl groups for transmethylation reactions that are essential for several biological processes (Figure 1). Methionine from dietary sources is converted to S-adenosyl methionine (SAM) by the enzyme S-adenosyl methionine synthase. The methyl group of SAM is required for over 100 known transmethylation reactions, including methylation of macromolecules, phospholipids, myelin, choline and catecholamine. During these reactions SAM is converted by various methyl transferases to S-adenosyl homocysteine (SAH), which is then hydrolyzed to homocysteine and adenosine by S-adenosyl homocysteine hydrolase. This is a reversible reaction with the equilibrium favoring the synthesis of SAH. Homocysteine once formed can either be remethylated to methionine by methionine synthase (MS) or betaine hydroxymethyl transferase (BHMT) and/or converted to cystathionine by cystathionine-beta-synthase (CBS). Excess homocysteine is exported into circulation where it rapidly binds to proteins and other small molecules like cysteine. In circulation < 1% of homocysteine is present in the free reduced form, while 10–20 % of the tHcy is present as homocysteine-cysteine mixed disulfide and homocystine (dimer of homocysteine), 80–90 % of homocysteine in circulation is protein bound [10]. The essential steps that contribute to the metabolism of homocysteine are outlined in (Figure 1). In healthy well nourished individuals homocysteine metabolism is well regulated and the plasma concentration is usually less than 12 μM. However, genetic defects or nutritional deficiencies lead to elevation of the levels of homocysteine.

Although hyperhomocysteinemia has been associated with several diseases, the mechanism of homocysteine-induced deleterious effects is not fully elucidated. Prominent among the various mechanism proposed for the harmful effects of homocysteine is its ability to modulate the expression of certain genes that may either directly or indirectly lead to several pathological conditions [11]. Homocysteine-induced modulation of gene expression may be due to altered methylation status as the levels of SAH, an inhibitor of many SAM-dependent methyl transferases (Mtase) are elevated during hyperhomocysteinemic conditions [12,13]. Apart from the modulation of gene expression due to altered methylation, homocysteine might modulate gene expression by hitherto unknown mechanisms [14].

## Methods

We manually screened all the abstracts from PUBMED, NCBI (up to November 2004) that contained the keywords "homocysteine" and "gene". The genes that are associated with homocysteine could be classified into two broad groups: (i) Genes that are modulated in response to elevated homocysteine levels (Table 1). Modulations of these genes are predicted to result in diseased states. (ii) Genes that are directly or indirectly involved in the modulation of homocysteine levels (Table 2). Defects in these genes, primarily due to single nucleotide polymorphism (SNP) have been, in some cases, shown to elevate the levels of homocysteine. The list was also verified using an in-house JAVA based text mining tool. We then mapped the genes in appropriate pathways/ networks, using databases that are available in the public domain, in an attempt to elucidate the probable mechanism of homocysteine induced deleterious effects.

**Table 1:** List of genes identified by literature mining that are modulated by elevated level of homocysteine

**S.NO**	**Symbol**	**Gene Name**	**Function**
1	Adk	Adenosine kinase	Methionine Metabolism
2	Agt1	Angiotensin I	Renin – Angiotensin
3	Ahcyl1	S-adenosylhomocysteine hydrolase – like	Methionine Metabolism
4	Bax	BCL2-associated × protein	Apoptosis
5	Bcl-2	B-cell cll/lymphoma 2	Apoptosis
6	Bhmt2	Betaine-homocysteine methyltransferase2	Methionine Metabolism
7	Calm1	Calmodulin 1	Signaling
8	Proxy1/Cap43	Protein regulated by oxygen 1	Hypoxia
9	Casp12	caspase-12	Apoptosis
10	Casp3	caspase-3	Apoptosis
11	Cav3	Caveolin	Apoptosis
12	Ccr2	Chemokine receptor 2	Atherosclerosis
13	Cdk2	Cyclin-dependent kinase 2	Apoptosis
14	Cetp	Cholesteryl ester transfer protein	Lipid metabolism
15	Cgrp	Calcitonin gene related peptide	Signaling
16	Cck	Cholecystokinin	Insulin secretion
17	Clu	Clusterin	Apoptosis
18	Cmyc	Myc proto-oncogene protein	Apoptosis
19	Cnp	C-type natriuretic peptide	Vasorelaxant activity.
20	Crp	C-AMP receptor protein	Apoptosis/signaling
21	Cubn	Cubilin	Vitamin B12 Transport
22	Cx43	Connexin43	Integral to plasma membrane/Signaling
23	Ccna1	Cyclin A1	Cell cycle
24	clcn	Chloride ion channel gene	Chloride transport
25	Cyc	Cytochrome	Apoptosis
26	Demethylase	Demethylase	Metabolism
27	Dhfr	Dihydrofolate reductase	Metabolism
28	Dnmt1	DNA Methyltransferase 1	Metabolism
29	Dnmt2	DNA Methyltransferase 2	Metabolism
30	Dnmt3a	DNA Methyltransferase 3	Metabolism
31	Erk2	Extracellular Signal-Regulated Kinase 2	Signalling
32	Fak	Focal adhesion kinase	Apoptosis
33	Fbp1/ Folr1	Folate-Binding Protein1	Folate transport
34	Fbp2	Folate-Binding Protein2	Folate transport
35	G6pdh	Glucose-6-phosphate dehydrogenase	Metabolism
36	Gad67	Glutamic acid decarboxylase 67	Apoptosis
37	Gadd153	Glutamic acid decarboxylase 153	Apoptosis
38	Gadd45	Glutamic acid decarboxylase 45	Apoptosis
39	Gata4	GATA-Binding Protein 4	Transcription factor
40	GPX1	Glutathione Peroxidase	Anti-oxidant
41	Gsh1	GS homeobox 1	Transcription Factor
42	Grp78	Glucose related protein 78	Apoptosis
43	Grp94	Glucose related protein 98	Apoptosis
44	H2B	Histone 2B	Histone protein
45	H3	Histone 3	Histone protein
46	HDACs	Histone deacetylases	Histone Deacetylation
47	Hmgcr	Hydroxy-3-Methylglutaryl-Coa Reductase	Lipid metabolism
48	Hmt	Homocysteine-S-methyltransferase	Metabolism
49	Ikβα	Inhibitor Of Kappa Light Chain Gene Enhancer	Signaling
50	IL-1	Interleukin 1	Signaling
51	IL-6	Interleukin 6	Signaling
52	IL-8	Interleukin 8	Signaling
53	Inmt	Indolethylamine N-methyltransferase	Protein methylation
54	iNOS	Inducible Nitric Oxide Synthase	Nitric oxide stress
55	Interferon	Interferon	Signaling
56	Ifg	Ifngamma	Signaling
57	Ldhd	D-Lactate Dehydrogenase	Metabolism
58	Ldlr	Low Density Lipoprotein Receptor	Lipid metabolism
59	Lpl	Lipoprotein lipase	Lipid metabolism
60	Lox1	Lectin like oxidized LDL receptor-1	Lipid Transport
61	Lpa	Apolipoprotein	Lipid metabolism
62	lyase	Lyase	Lipid metabolism
63	Mcp1	Monocyte Chemoattractant Protein 1	Atherosclerosis
64	Mbd2	Methyl-CpG-Binding Domain Protein 2	Methylation binding protein
65	Mecp2	Methyl-CpG-Binding Protein 2	Methylation binding protein
66	Mapk/Mek	Mitogen-Activated Protein Kinase Kinase	Signalling
67	Mgmt	O6-methylguanine-DNA methyltransferase	Apoptosis
68	Mmp3	Matrix metalloproteinase 3	Remodeling of extracellular matrix
69	Mtap	Methyl Thioadenosine Phosphorylase	Metabolism
70	Mtase	Methyltransferase	Metabolism
71	NF-Kβ	Nuclear Factor Kappa-B	Signaling
72	Nmda	N-methyl-D-aspartate receptors	Alzheimer Disease
73	Nos2	Nitric Oxide Synthase 2	Nitric oxide Synthesis
74	P21 ras	P21 ras	Signaling
75	P38	Serine /threonine protein kinase belong to MAPK subfamily	Apoptosis
76	P53	Tumor protein p53	Apoptosis
77	Pai-1	Plasminogen Activator Inhibitor-1	Blood coagulation
78	Pam	Peptidylglycine alpha-amidating monooxygenase	Neuro peptide amidation
79	Icmt/Pcmt	Isoprenylcysteine Carboxylmethyltransferase	Signaling.
80	Pdgf	Platelet-derived growth factor	Inhibits apoptosis
81	Pemt	Phosphatidylethanolamine (PE) N-Methyltransferase	Methylation of PE
82	Pkc	Protein kinase C	Apoptosis
89	Ppar alpha	Peroxisome Proliferator-Activated Receptor-Alpha	Signaling
84	PPARgamma2	Proliferator-Activated Receptor-Gamma2	Signaling
85	Prmt	Protein Arginine N-Methyltransferase	Protein methylation
86	Ps1	Presenilin 1	Alzheimer Disease
87	S3a	Ribosomal protein S3A	Structural constituent of Ribosome
88	Smap8	smooth muscle-associated protein 8	Signaling
89	Srebp1	sterol regulatory element binding protein-1	Lipid Transport
90	Sst	Somatostatin	Alzheimer Disease
91	Tdag51	T-cell death-associated gene 51	Apoptosis
92	TGFbeta	Transforming growth factor beta	Apoptosis
93	TNFalpha	tumor necrosis factor alpha	Signaling
94	TNFRSF1B	Tumor necrosis factor receptor 2 gene	Signaling
95	Timp1	Tissue Inhibitor Of Metalloproteinase 1	Signaling
96	tPA	Tissue-type plasminogen activator	Blood Coagulation
97	Vcam 1	Vascular Cell Adhesion Molecule 1	Cell adhesion/Signaling
98	Yy1	Yin Yang 1	Transcription factor
99	F2	Coagulation factor II	Blood Coagulation
100	HemK/PrmC	N5-glutamine AdoMet-dependent methyltransferase	Methylation
101	ABCC2	ATP-Binding Cassette subfamily C	Cellular cisplatin transporter.
102	Ace	Angiotensin converting enzyme	Renin – Angiotensin
103	Nat1	arylamine N-acetyltransferase type-1	Detoxification of a plethora of hydrazine and arylamine drugs
104	Gnmt	Glycine N-Methyltransferase	Methylation
105	Apo B	Apolipoproteine B	Lipid metabolism
106	Ins	Insulin	Signalling
107	Sod	Super Oxide Dismutase	Anti-oxidant
108	ApoC3	Apolipoprotein C-III	Lipid metabolism
109	Atf3	Activating transcription factor	Transcription factor
110	Ap1	activating protein-1	Transcription factor
111	Fcmt	Farnesylcysteine methyltransferase	Methylation
112	Hmox	Heme oxygenase	Biliverdin metabolism

**Table 2:** List of genes identified by literature mining that modulate homocysteine levels

**S.No**	**Symbol**	**Gene Name**	**Function**
1.	Mthfr	Methylenetetrahydrofolate Reductase	Conversion of 5, 10-methylene-tetrahydrofolate to 5-methyl-tetrahydrofolate.
2.	Cbs	Cystathionine beta-synthase	Condensation of homo-cysteine and serine to form cystathionine
3.	Mtr	Methyltetrahydrofolatehomocysteine methyltransferase	Remethylation of homocysteine to methionine
4.	Mtrr	Methionine synthase reductase	Reductive regeneration of cob(I)alamin cofactor required for the maintenance of MTR in a functional state
5.	Rfc-1	Reduced-folate carrier	5-methyl-tetrahydrofolate internalization in cell
6	Gcp II/Folh1	Glutamate Carboxypeptidase II	Polyglutamate converted to monoglutamate folate by action of the enzyme folylpoly gammaglutamate carboxy-peptidase (FGCPI), an enzyme expressed by GCPII.
7	eNos	Endothelial Nitric oxide synthase	Conversion of L-Arginine to L-Citrulline and nitric oxide synthase (NO)
8.	Tc II	Transcobalamine II	Transport of vitamin B12
9.	Shmt1	Serine Hydroxymethyltransferase 1	Reversible conversion of serine and tetrahydrofolate to glycine and 5, 10-methylene tetrahydrofolate.
10.	Tyms	Thymidylate Synthase	5, 10-methylene THF and deoxyuridylate to form dihydro-folate and thymidylate.
11	Cth	Cystathionine Gamma-Lyase	Hydrolysis of cystathionine to cysteine and α-Ketoglutarate
12	Mthfd	Methylene-tetra hydrofolate dehydrogenase	Conversion of 5, 10-methylene-tetrahydrofolate to5, 10methenyl-tetrahydrofolate.
13	Mthfs	Methenyltetrahydrofolate synthetase	Conversion of 5-formyltetrahydrofolate to 5, 10-methenyltetrahydrofolate.
14	Apo E	Apolipoproteine E	Mediates the binding, internalization, and catabolism of lipoprotein particles.
15	Vegf	Vascular endothelial growth factor	Growth factor active in angiogenesis, vasculogenesis and endothelial cell growth.
16	Pon1	Paraoxonase 1	Hydrolyzes the toxic organo-phosphorus. It also mediate an enzymatic protection of LDL against oxidative modification.
17	Bhmt	Betaine-homocysteine methyltransferase	In Liver & kidney it catalyses the conversion of betaine to dimethyl glycine (DMG).
18	Mat1A	Methionine Adenosyltransferase 1A	Methionine to SAM by transfer of the adenosyl moiety of ATP to the sulfur atom of methionine
19	Ahcy	S-adenosylhomocysteine hydrolase	Hydrolysis of AdoHcy to adenosine and homocysteine
20	Cbl	Cystathionine beta lyase	Conversion of cystathionine to homocysteine.
22	Factor V	Coagulation factor V	Cofactor for the factor Xa-catalyzed activation of prothrombin to the clotting enzyme thrombin.
23	Pai-1	Prothrombin activator inhibitor-1	Inhibition of fibrinolysis by inhibiting the plasminogen-activator and t-PA.

## Physiological processes that are affected due to homocysteine-induced modulation of gene expression

### Elevated homocysteine levels and oxidative stress

**Figure 2:**
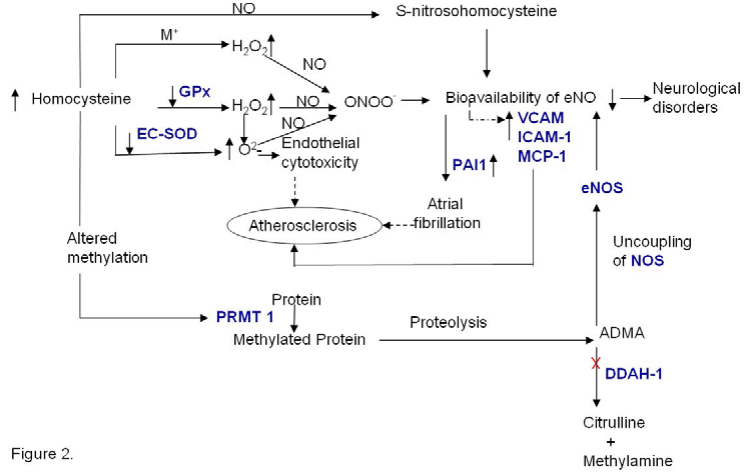
**Hyperhomocysteinemia and Oxidative Stress**. Homocysteine might directly or indirectly lead to oxidative stress via the pathways shown in the figure. The genes marked in blue have been identified by literature based searches as mentioned in the methods section. The solid and dotted lines indicate direct and indirect (multi step) interaction/ conversion respectively. **X **– Inhibition

One of the mechanisms proposed for the deleterious effects of homocysteine is its ability to generate reactive oxygen species thereby producing oxidative stress (Figure 2). It is generally proposed that homocysteine, due to the presence of a thiol group, can rapidly auto-oxidize in circulation in the presence of ceruloplasmin, the major copper binding protein in plasma, to form homocystine and hydrogen peroxide (H_2_O_2_), thereby generating oxidative stress [15]. However, several recent reports indicate that transition metal catalyzed oxidation of homocysteine is not a facile process. In fact transition metal catalyzed oxidation of cysteine has been reported to be much faster than that of homocysteine [16] and although the concentration of cysteine is about 20–25 times higher than that of homocysteine it is usually not considered a risk factor for cardiovascular diseases [17]. Therefore, it seems unlikely that the deleterious effect of homocysteine is due to the generation of hydrogen peroxide via metal catalyzed auto-oxidation. However, homocysteine might indirectly result in oxidative stress by decreasing the transcription, translation [18] and catalytic activity of antioxidant enzymes like glutathione peroxidase (GPx) and superoxide dismutase (SOD) [19]. Homocysteine treated bovine aortic endothelial cells showed a significant decrease in glutathione peroxidase activity. The effect of Homocysteine on enzyme activity is demonstrated by compensatory effect of GPx-1 over expression on the adverse effects of homocysteine on endothelial function [20]. Nonaka et al [21] reported that homocysteine decreases the secretion and expression of extra cellular superoxide dismutase (EC-SOD), the most abundant isozyme of SOD, in the vascular wall in rat vascular smooth muscle cells.

Hyperhomocysteinemia has also been reported to be associated albeit indirectly with hypoxic conditions. Supporting this is the expression of Cap43 [that codes for a 43 kDa protein associated with hypoxia in endothelial cells (EC)] in cells treated with homocysteine. Hypoxia in alveoli leads to damage of capillary wall, a condition predisposing for atherosclerosis. Furthermore, it has also been shown that there is a decrease in the MAT1A transcription and mRNA stability in cultured hepatocytes exposed to hypoxic conditions [22].

Elevated levels of homocysteine have been reported to decrease the bioavailability of endothelial nitric oxide. Under normal condition Nitric oxide (NO) exerts anti-atherosclerotic effect through various mechanisms (Table 3). The NO produced by endothelium is known to decrease in response to elevated levels of homocysteine [23]. Decrease in the bioavailability of nitric oxide due to increased homocysteine concentration is perceived to cause vasoconstriction thus leading to cardiovascular disorders. Interestingly, the bioavailability of NO is decreased in hyperhomocysteinemic condition despite normal expression of eNOS [24]. The decrease in the bioavailability of nitric oxide in hyperhomocysteinemic conditions may be attributed to the formation of S- nitrosohomocysteine formed under physiological conditions (Figure 2). NO can also rapidly react with molecular oxygen and other oxygen free radicals to form peroxynitrites (ONOO^-^) [25-27]. Furthermore, endothelial tetrahydrobiopterin, a critical co-factor for the endothelial nitric-oxide synthase (eNOS) is also a target for oxidation by ONOO^- ^and its oxidation results in formation of trihydrobiopterin radical (BH3^•^) and consequently decrease NO production [28,29]. Thus, elevated levels of homocysteine may lead to the accumulation of reactive oxygen species due to the decreased activity of antioxidant enzymes and these oxygen radicals could then potentially inactivate NO resulting in vasoconstriction.

**Table 3:** Mechanisms mediating the anti-atherosclerotic effect of nitric oxide

**Anti-atherosclerotic effect of nitric oxide**	**Reference(s)**
Promotion of SMC proliferation	131
Inhibition of platelet aggregation	132
Reduction in endothelial activation & Inhibition of MCP-1	133, 134
Stabilizes NF-Kβ inhibitor, Ikβα	135
Inhibition of LDL oxidation & lipid peroxidation	136,137
Reduces super oxide generation	138
Decrease the Expression of PAI-1	34
Nitric oxide regulates vascular cell adhesion molecule 1 gene expression	139

Another potential mechanism for the decreased bioavailability of NO in hyperhomocysteinemic states is the increased generation of asymmetric dimethylarginine (ADMA), an analogue of L-arginine, which is a competitive inhibitor of eNOS [30]. ADMA also promotes the "uncoupling" of eNOS (Figure 2) leading to increased production of superoxide & other reactive oxygen species, which may cause further decrease in availability of NO. ADMA is produced during degradation of proteins containing methylated arginine residues by protein arginine N- methyltransferases (PRMTs) [31]. The increased SAM dependent generation of these methylated proteins, results in both increased production of ADMA and increased generation of homocysteine [32]. The inhibition of endothelial nitric oxide synthesis by ADMA impairs cerebral blood flow, which may contribute to the development of Alzheimer's disease [33].

Furthermore, one of the mechanisms proposed for the anti-thrombotic effect of NO is its ability to inhibit the expression of the prothrombotic protein PAI-1. It has also been shown that NO released from activated platelets inhibits the recruitment of platelets to the growing thrombus [34]. Thus decrease in NO concentration may result in increased expression of PAI-1 and platelet aggregation leading to thrombosis.

Intracellular oxidative stress may be either due to excessive generation of reactive oxygen species or to decreased ability of cells to scavenge the reactive oxygen species leading to its accumulation. We propose that homocysteine-induced oxidative stress is primarily due to the decreased ability of the cells to detoxify H_2_O_2 _& other lipid peroxides due to decreased activity of intracellular antioxidant enzymes. Furthermore, decreased bioavailability of nitric oxide may lead to the increased expression of pro-inflammatory cytokines and PAI which can potentially lead to cardiovascular diseases.

### Hyperhomocysteinemia: apoptosis and inflammatory pathways

**Figure 3:**
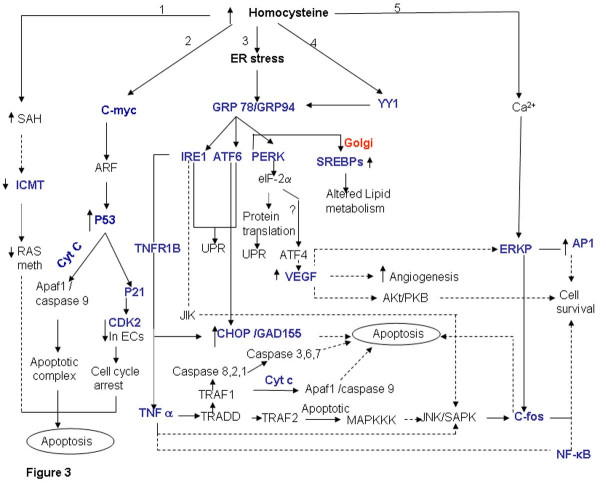
**Elevated homocysteine levels and apoptosis**. Elevated levels of homocysteine directly (branch 1 and 2) or via ER stress (branch 3 and 4) lead to apoptosis. Homocysteine also might lead to cell survival via calcium dependent ERK phosphorylation (branch 5). The genes marked in blue have been identified by literature based searches as mentioned in the methods section. The solid and dotted lines indicate direct and indirect (multi step) interaction/ conversion respectively.

The major process linking levels of homocysteine with apoptosis and inflammatory pathway is the Endoplasmic Reticulum (ER) stress (Figure 3, branch 3,) and c-myc mediated signaling (Figure 3, branch 2). Endoplasmic Reticulum is the destination for secretary and extracellular proteins. It also serves as a site of calcium storage, calcium signaling, and biosynthesis of steroids, cholesterol & other lipids. The ER has high level of numerous resident chaperone proteins such as glucose-regulated proteins, GRP-78 & GRP-94, which under normal conditions are required for proper protein folding prior to export, to their destination. However, during energy deprivation these proteins initiate signal of ER stress, a condition in which unfolded & misfolded proteins accumulate [35,36]. Homocysteine has been shown to alter the cellular redox state resulting in ER stress [37]. Homocysteine increases the expression and synthesis of GRP78, a glucose-regulated protein that is induced during ER stress. Cells respond to ER Stress by various processes, prominent among these is a process known as unfolded protein response (UPR) mediated by ER-resistant trans-membrane protein kinase (IRE1) [38]. Furthermore, Homocysteine was found to induce the expression of glutamic acid decarboxylase (GADD45, GADD 153), ATF4 (Figure 3, branch 3) and YY1 [[38], Figure 3, branch 4] as well as RTP and HERP [39,40]. Interestingly, in a separate study it has been reported that inducers of GRP78 also increase the expression of these genes [41]. The GADDs also link homocysteine levels with ER stress and alterations in cell growth and proliferation. In addition, YY1, a member of GL1 zinc finger family enhances the transcription activation of GRP78 promoter under a variety of ER stress conditions [42,43]. Increased expression of YY1 mediates the stress signal from ER to nucleus.

However, exposure to excess ER stress results in apoptotic cell death. ER stress activates c-Jun N-terminal kinases (JNKs) that regulate gene expression via phosphorylation and activation of transcription factors such as c-JUN. The activation of JNK is mediated by TNF receptor-associated factor-2 (TRAF2), which transduce signals from IREs that act as stress sensors and initiates UPR [44]. TRAF2 activates the apoptosis-signaling kinase (ASK1) or MAPKKK (mitogen activated protein kinase kinase kinase). Activation of MAPKKK leads to activation of JNK protein kinase that in turn causes apoptosis [45]. The TRAF1 binds to the TRADD (TNFR-Associated Death Domain), which recruits the activated caspase 8 initiating a proteolytic cascade subsequently resulting in apoptosis. Furthermore, caspase 8 also leads to release of pro-apoptotic factor cytochrome C [46]. Homocysteine may induce oxidative stress and apoptosis through an NADPH oxidase and/or JNK-dependent mechanism(s) [47]. Extra cellular adenosine (Ado) along with homocysteine (Ado/Hcy) causes apoptosis of cultured pulmonary artery endothelial cells through the enhanced formation of intracellular S-adenosylhomocysteine (Figure 3, branch 1). SAH inhibits isoprenylcysteine carboxylmethyltransferase (ICMT), which results in decrease of Ras methylation and activation of downstream signaling molecules resulting in apoptosis. ICMT catalyzes the posttranslational methylation of isoprenylated C-terminal cysteine residues found in many signaling proteins such as small monomeric G proteins [48,49]. Similarly high concentration of adenosine results in apoptosis of L1210 lymphocytic leukemia cells. Apoptosis in these cells was preceded by an early but transient expression of the proto-oncogene c-myc [50].

Expression of c-myc sensitizes cells to a wide range of pro-apoptotic insults that include DNA damage, hypoxia and nutrient deprivation (Figure 3, branch 2). The pro -apoptotic effect of c-myc is mediated through the release of cytochrome C into the cytosol [51]. Holocytochrome C interacts with apoptotic protease activating factor (APAF-1), which then recruits and activates procaspase 9. This ternary complex triggers the autocatalytic processing of caspase 9 and subsequently activates caspase 3. However inhibition of CD95 and P53 signaling pathway does not block this release, but activation of the caspase dependent apoptotic machinery requires cooperation between c-myc induced cytochrome C release and CD95 signaling. Thus, c-myc induction leads to release of cytochrome C to the cytosol recruiting the cells to other apoptotic triggers like CD95 pathway or p53 activation. C-myc might also induce apoptosis through active response factor (ARF) expression via activation of P53 [52]. Recently it has been shown that homocysteine induced apoptosis in human umbilical vein endothelial cells is correlated with p53 dependent Noxa expression [53]. The expression of the Noxa gene involves direct activation of its promoter by p53. Interestingly, the activity of p53 is regulated through lysine methylation. Methylated p53 is restricted to the nucleus and has increased stability. The "hyper-stabilization" and activation of p53 result in cell cycle arrest and apoptosis [54]. The methyltransferase activity is critical for p53 dependent apoptosis. Thus, it can be perceived that in hyperhomocysteinemic state p53 lysine methylation could be inhibited. Homocysteine could also potentially inhibit endothelial cell growth by inhibiting the expression of cyclin A mRNA. Apart from cyclin A associated kinase activity, cyclin dependent kinase (CDK2) activity was also significantly inhibited [55]. Interestingly, stress induced activation of P53 promotes transcription of P21, which in turn binds to CDKs and leads to blocking of the G1 to S phase transition during cell cycle.

Homocysteine affects mitogenesis in a cell type specific manner. Although elevated levels of homocysteine lead to apoptosis and has growth inhibitory effect on endothelial cells, it leads to proliferation of smooth muscle cells eg. homocysteine enhances AP-1 activity in A7r5 aortic smooth muscle cells thus influencing cell proliferation [56]. In a recent report it was shown that elevated levels of homocysteine result in increased AP-1 nuclear protein binding, cell DNA synthesis and proliferation in mesangial cells by increasing Erk activity via a calcium-dependent mechanism [[57], Figure 3, branch 5]. Furthermore, homocysteine has been reported to up regulate the expression of VEGF mRNA in pigmented human endothelial cell line via ATF4 mediated activation. [58]. The cell survival signal from VEGF is mainly brought about by P13-mediated activation of Akt/PKB. The downstream targets for Akt/PKB pathway inhibit apoptosis. Furthermore, VEGF also leads to the induction of Raf-MEK -ERK pathway in human umbilical endothelial cells (HUVECs) relating to cell survival [59].

Apart from activating the unfolded protein response, homocysteine-induced ER stress also activates the sterol regulatory binding proteins (SREBPs). Homocysteine induces the expression of sterol regulatory element binding protein-1 (SREBP1, Figure 3), an ER membrane bound transcription factor, in cultured vascular endothelial cells and human hepatocyte leading to increased biosynthesis and uptake of cholesterol, triglycerides and accumulation of intracellular cholesterol [60,61]. Normally the expression and activity of SREBPs is regulated by SREBP cleavage activation protein (SCAP). However, it is believed that homocysteine circumvents this mechanism, maintaining the cells in sterol-starved state although lipids continue to accumulate.

Thus by mapping the genes (identified using literature based search) in appropriate pathway, we show that elevated levels of homocysteine cause the up regulation of ER stress proteins resulting in apoptosis. Homocysteine might also mediate apoptosis via P53 mediated pathway or by inhibition of methyl transferases like ICMT. Furthermore, ER stress also leads to altered lipid metabolism which may lead to cardiovascular disorders. Thus, homocysteine-induced ER stress emerges as the common pathway that relates to apoptosis and atherosclerosis. In this context it needs to be mentioned that homocysteine can potentially cleave critical protein disulfide bonds resulting in the alteration of structure and/or function of the protein [62-64]. It can be perceived that this might also lead to protein misfolding /unfolding which is a hallmark of ER stress.

### Hyperhomocysteinemia and the coagulation cascade

**Figure 4:**
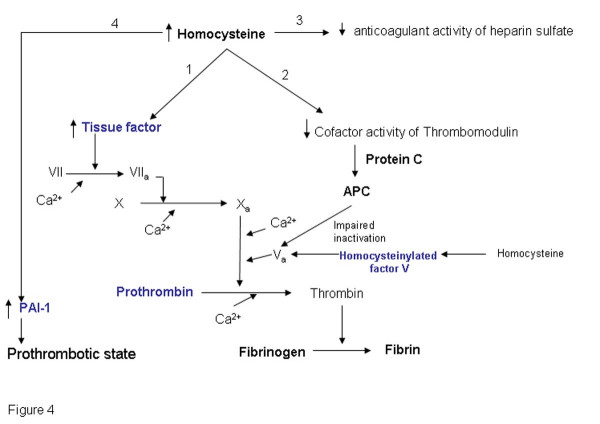
**Elevated homocysteine levels and the Coagulation pathway**. Elevated homocysteine levels may lead to thrombosis either by increasing the activity of the tissue factor (branch 1) thereby facilitating the coagulation cascade or by inhibiting the anticoagulant pathways (branch 2 and 3). The genes marked in blue have been identified by literature based searches as mentioned in the methods section. The solid and dotted lines indicate direct and indirect (multi step) interaction/ conversion respectively.

During vascular injury, tissue factor, an integral membrane glycoprotein that is tightly associated with phospholipids, form a complex (1:1) with factor VII thereby initiating the coagulation cascade (Figure 4). Homocysteine can enhance the pro-coagulant activity in a number of ways. Elevated homocysteine levels have been reported to increase the cellular tissue factor activity [65]. Mann et al [63] also suggested that homocysteine rapidly incorporates into factor V resulting in impaired inactivation of factor Va by activated protein C (APC). Binding of homocysteine to factor V however did not have any effect on the conversion of factor V to factor Va. APC, a vitamin K dependent protein is formed by the action of thrombin on protein C in the presence of a membrane bound cofactor, thrombomodulin. Interestingly, homocysteine has also been shown to inhibit the cofactor activity of thrombomodulin [66]. Thus homocysteine impairs the thrombomodulin-APC anticoagulant pathway by inhibiting the cofactor activity of thrombomdulin resulting in the decreased formation of APC and also by inhibiting the inactivation of factor Va by APC. Furthermore, homocysteine also affect another important endothelial anticoagulant pathway viz. endothelial cell heparin-like glycosaminoglycans-antithrombin III anticoagulant mechanism [67]. It has been reported that incubation of endothelial cells with homocysteine suppresses the amount of antithrombin III binding to cell surface. Homocysteine also reduces the cellular binding for tissue plasminogen activator and enhances the plasminogen activator inhibitor-1 (PAI-1) gene expression and secretion from vascular endothelial and smooth muscle cells by a mechanism independent from paracrine-autocrine activity of TGF beta and TNF alpha. Hajjar et al also suggested that endothelial cells treated with homocysteine resulted in selective reduction in cellular binding sites for t-PA. This 65% decrease in binding was associated with a 60% decrease in cell-associated t-PA activity [64]. Thus homocysteine may promote prothrombotic state.

Thus, it can be perceived that elevated homocysteine levels will lead to prothrombotic state by enhancing the pro-coagulant pathway and/or suppressing the anticoagulant pathways.

### Hyperhomocysteinemia and atherosclerosis

**Figure 5:**
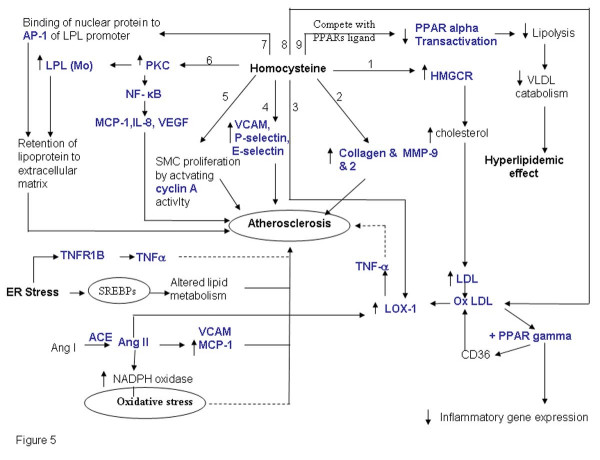
**Elevated Homocysteine is associated with atherosclerosis**. Elevated level of homocysteine affects the cholesterol biosynthesis (branch 1), expression of extracellular matrix proteins (branch 2), enhance endothelial LOX-1 gene expression and TNFα release upon oxLDL stimulation (branch 3,branch 8), affect the expression of cell adhesion molecule (branch 4), enhance SMC proliferation by increasing cyclin A activity (branch 5), induce the expression of LPL both at the transcription and translation level presumably via PKC activation (branch 6,7) that modulates inflammatory gene response in endothelial cells. Elevated level of homocysteine also down regulate the expression of PPARs (Figure 6, branch 9). The genes marked in blue have been identified by literature based searches as mentioned in the methods section. The solid and dotted lines indicate direct and indirect (multi step) interaction/ conversion respectively.

Atherosclerosis is a chronic inflammatory disease of the artery, in which deposits of fatty substances, cholesterol, cellular waste products, calcium and other substances build up in endothelial layer of artery [68]. Apart from the conventional risk factors for atherosclerosis, elevated level of homocysteine is now considered to be an independent risk factor for cardiovascular diseases. Several mechanisms have been proposed for the homocysteine induced cardiovascular disease including altered lipid metabolism, cholesterol dysregulation, modulation of extracellular matrix protein expression, inflammatory response and oxidative stress. From the genes obtained after literature mining we found that homocysteine might induce atherosclerosis via one or more of the pathways depicted in Figure 5. The various interlinked pathways that contribute to the complex phenotype of atherosclerosis are outlined below.

**Figure 6:**
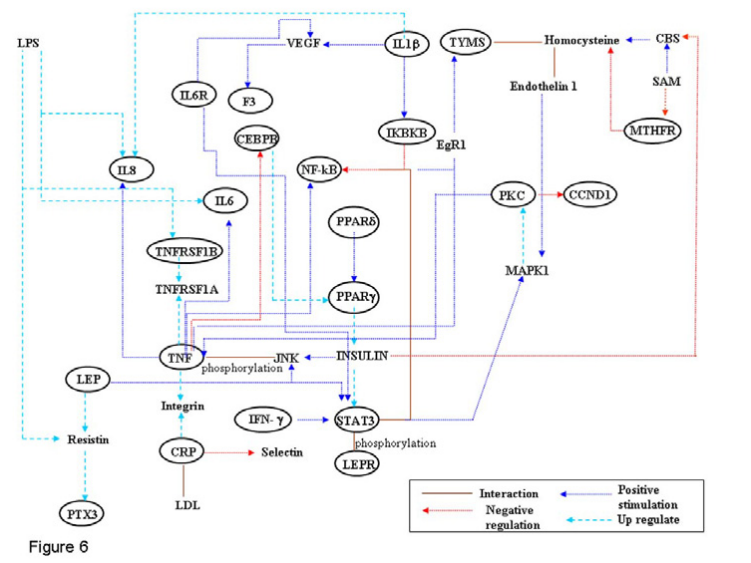
**Interactions between proinflammatory cytokines**. The interaction between different proinflammatory cytokines is shown which may be modulated by homocysteine levels resulting in pathological consequences.

#### I) Homocysteine mediates cholesterol dysregulation

Homocysteine plays an important role in cholesterol biosynthesis by inducing the transcription as well as translation of 3-hydroxy-3- methylglutaryl coenzyme A reductase (HMGCR), the rate-limiting enzyme in the cholesterol biosynthesis (Figure 5, branch 1). It also increases cholesterol synthesis and accumulation in endothelial cells [69]. Inhibitors of HMGCR like simvastatin prevented the homocysteine-induced accumulation of cholesterol. Thus, it can be perceived that elevated levels of homocysteine result in cholesterol biosynthesis dysregulation. Furthermore, as mentioned earlier, sterol regulatory element-binding protein-2 (SREBP), a transcription factor, is activated in the liver of hyperhomocysteinemic rats and the activation of SREBP-2 leads to hepatic lipid accumulation by regulating HMG-CoA reductase expression in the liver [70]. Hyperhomocystenemia also modulates cholestrol biosynthesis pathway through upregulation of the ER chaperone, GRP78/BiP in hepatocytes while the actual transport of the cholesterol in endothelial cells was found to be downregulated leading to upregulation of HMGCR in endothelial cells [71].

#### Ii) Homocysteine affects LPL and Lox-1 expression, which leads to atherosclerosis

Homocysteine has been found to induce the expression of macrophage lipoprotein lipase (LPL) both at the transcription and translation level presumably via PKC activation [[72], figure 5, branch 6]. LPL is the major lipolytic enzyme involved in hydrolysis of triglycerides in lipoproteins [73]. It is secreted by macrophages in atherosclerotic lesions and macrophage LPL produced in the vascular wall acts as a pro-atherogenic protein. This enzyme mediates the uptake of lipoproteins by macrophages, promotes lipoprotein retention to the extracellular matrix, induces the expression of the proatherogenic cytokine TNF-α, increase monocyte adhesion to endothelial cells and proliferation of vascular smooth muscle cells. It also promotes foam cell formation and atherosclerosis in vivo. Homocysteine was found to simultaneously change macrophage LPL & c-fos mRNA levels and induce the binding of nuclear protein to AP1 sequence (Figure 5, branch 7). This suggests that c-fos also may have a role to play in the stimulatory effect of homocysteine on macrophage LPL mRNA expression.

Homocysteine is known to down regulate the expression of peroxisome proliferators-activated receptors (PPARs) that are redox sensitive transcription factors in the vasculature belonging to the ligand-activated nuclear receptor family (Figure 5, branch 9). They play a key role in regulating expression of genes that control glucose and lipid metabolism and has been implicated in metabolic disorders leading to atherosclerosis. PPAR agonists like fibrates are known to promote anti-inflammatory effects presumably via the induction of antioxidant enzymes by PPARs. Homocysteine can potentially bind to PPARs and compete with the PPAR ligands like fibrates [74]. In fact it has been reported that homocysteine binds to PPARs with a 10 fold higher affinity than fibrates [74], a class of lipid-modifying agents that have been widely used to substantially decrease plasma triglyceride levels. It also results in moderate decrease in LDL cholesterol and an increase in HDL cholesterol concentrations. Thus, elevated levels of homocysteine might lead to hyperlipidemia by competing with the PPAR ligands like fibrates which are known to catabolise VLDL and triglycerides [75].

Oxidized low density lipoprotein (OxLDL) (Figure 5, branch 8) is one of the major factor that is responsible for endothelial dysfunction is as it induces expression of adhesion molecules, chemokines like MCP1 and impairs the endothelium-dependent vasorelaxation. LOX-1 is the principle receptor of OxLDL in vascular endothelial cells. Homocysteine has been reported to enhance endothelial LOX-1 gene expression and TNFα release upon oxLDL stimulation [[76,77] Figure 5, branch 3]. Tontonoz et al demonstrated that oxLDL induces PPAR-γ in foam cell of atherosclerotic lesion, thus potentiating pathogenesis of atherosclerosis [78]. Oxidized LDL has a role in the activation of PPAR-γ dependent gene expression and regulation of oxLDL receptor CD36. Thus, activation of PPAR-γ and CD36 constitute a positive feedback loop to potentiate the effects of oxLDL. PPARα and PPAR-γ can also suppress the inflammatory gene expression in monocytes, [79,80] and mediate the anti-inflammatory response in the vessel wall. Hence, a balance between pro inflammatory effect of oxLDL and anti-inflammatory properties of PPARs determine the inflammatory status of cell/ vessel wall. Recent cross sectional studies report that oxidized LDL have higher association with angiographically documented coronary artery disease in patients 60 years or younger which implies that early onset CAD is more correlated with oxidized LDL thus by upregulating oxidized LDL receptors homocysteine induces athreosclerotic changes in an independent manner in both endothelial cells as well as mononuclear cells accelerating the rate of atherosclerosis [81].

#### (iii) Homocysteine modulates inflammatory gene response in endothelial cells

In endothelial cells, proinflammatory cytokines enhance the binding of NF-κB to DNA and cause up-regulation of NF-κB dependent genes [82,83] (Figure 5, branch 6) Homocysteine has been reported to induce NF-κB activation in HUVECs and human aortic endothelial cells (HAECs). It also activates IκB-α resulting in nuclear translocation of NF-κB and enhanced NF-κB /DNA interaction. Thus, homocysteine cause an imbalance in intracellular signaling rather than a complete suppression of endothelial cell function. NF-κB may also play an important role in homocysteine-induced MCP-1 expression leading to monocyte macrophage accumulation in atherosclerotic lesions. Wang et. al [84] demonstrated that in homocysteine treated vascular smooth muscle cells both mRNA and protein levels of MCP-1 were increased through activation of PKC and superoxide production followed by NF-κB activation. MCP-1 & IL-8 are major chemokines for leukocyte trafficking and has been found in atheromatous plaques. The major route of action of MCP-1 is via its interaction with MCP-1 receptor on surface of monocyte (CCR2). Homocysteine stimulates CCR2 expression in monocyte leading to an enhanced binding and chemotactic response [84]. Apart from MCP-1, homocysteine also up regulate the expression of IL-8 in cultured human monocyte via enhanced formation of homocysteine induced ROS (Reactive Oxygen species) [85]. Homocysteine also induces expression of VEGF presumably via activation of NF-κB [86]. VEGF has been found to be expressed in activated macrophages, endothelial cells, and smooth muscle cells in human coronary atherosclerotic lesions, but not in normal artery [87]. VEGF has also been reported to increase atherosclerotic plaque size [88].

Moreover, in endothelial cells homocysteine modulates the expression of cell adhesion molecule-1 (sCAM-1) [89] which is generally perceived as a marker for vascular inflammation. Mansoor et. al. recently reported increase in the concentration of plasma homocysteine and triglycerides six hours after methionine and/or fat loading. It resulted in significant increase in the concentrations of P-selectin, E-selectin and VCAM-1 in healthy volunteers [90](Figure 5, branch 4).

Increasing evidence suggests the role of hyperhomocysteinemia in the underlying pathophysiological mechanism of the increased vascular risk development of coronary artery disease in patients with T2DM (Type 2 Diabetes Mellitus). The mechanisms by which homocysteine promotes this and exerts its detrimental effects may relate to induction of endothelial dysfunction and/or chronic inflammation (Figure 5, branches 4–6). T2DM stems from the failure of the body to respond normally to insulin, called "insulin resistance", ultimately leading to hyperglycemic condition. This common form of diabetes is often associated with obesity. Studies on experimental models have suggested that obesity also is a state of chronic inflammation. Over the years increasing evidence has accumulated indicating an ongoing cytokine-induced acute-phase response (low-grade inflammation) to be closely involved in the pathogenesis of T2DM and associated complications such as dyslipidemia and atherosclerosis. Observation that plasma concentrations of proinflammatory markers viz. C-reactive protein (CRP), interleukin-6 (IL-6), plasminogen activator inhibitor-1 (PAI-1) and tumor necrosis factor-α (TNF-α) in the obese are elevated has confirmed the same. The interactions between the proinflammatory cytokines are shown in (figure 6). Association of hyperhomocysteinemia with elevated levels of proinflammatory cytokine in T2DM patients substantiates its role in accelerating diabetes associated atherosclerosis [91]. Impaired SAM synthesis in liver tissue has also been shown to enhance production of pro-inflammatory cytokines and mediators [92].

#### V) Hypertension, angiotensin II and atherosclerosis

Hypertension is a risk factor for cardiovascular disease, and experimental evidence supports a role of renin-angiotensin system in contributing to pathogenesis of atherosclerosis [[93], Figure 5]. Untreated hypertension is associated with disturbed glutathione redox status and increased plasma homocysteine concentrations [94]. Hypertension associated with the elevation of angiotensin II levels results in the induction of smooth muscle cell superoxide via NADPH oxidase [95,96]. In addition, angiotensin II has also been shown to stimulate MCP-1 and VCAM -1 expression in rat aorta [97] and elevate LOX-1 expression in cultured vascular endothelial cells [98]. Interestingly, homoctysteine as mentioned above induces the expression of MCP-1, VCAM-1 and LOX-1 [77,85]. Thus, elevated levels of homocysteine may lead to hypertension by mechanisms similar to that of angiotensin II. This hypothesis is further supported by the report that methionine loading in normotensive and spontaneously hypertensive rats resulted in quantitative difference in homocysteine in the two rats. In spontaneously hypertensive rat, the serum levels of homocysteine were higher than in normotensive rats. Furthermore, methionine-related aortic alterations developed earlier were considerably more pronounced with the formation of additional connective tissue in spontaneously hypertensive rats [99]. Interestingly, administration of angiotensin II exacerbated the methionine loading-related aortic alterations. Mild hyperhomocysteinemia is associated with stiffer small arteries with increased collagen deposition but these changes are accentuated by angiotensin II-induced blood pressure elevation [100]. There is also a report, which suggests that in NIH/3T3 fibroblasts, angiotensin II induces GATA4 activity and homocysteine delayed this binding and hence alters the angiotensin II signaling [101]. It is thus perceived that the deleterious effects of homocysteine may at least in part be mediated via modulation of angiotensin II -signaling for gene transcription.

#### vi) Homocysteine and extra cellular matrix

Homocysteine up regulates the synthesis and accumulation of SMC collagen [[102] Figure 5, branch 2] and several studies have demonstrated that homocysteine is mitogenic for arterial SMCs [103,104] Extra-cellular matrix proteins like collagen are known to be critical components of atherosclerotic lesions [105]. The proliferation of smooth muscle cells and synthesis of extracellular matrix are important determinants of the extent of lesion development and plaque stability. Fibrillar collagen has an important role in the pathogenesis of atherosclerosis due to its substantial contribution to the mass of connective tissue. It renders structural support for the plaques [106]. Uncontrolled collagen accumulation leads to arterial stenosis, while excessive collagen breakdown combined with inadequate synthesis weakens plaques thereby making them prone to rupture.

Apart from collagen, homocysteine induces matrix metalloproteinases. Remodeling of extra-cellular matrix of the arterial wall by inducing elastolysis via activation of metalloproteinases in response to elevated levels of homocysteine is shown by studies in animal models. Chaussalet et al [107] showed that pathological levels of homocysteine increased the secretion of elastolytic metalloproteinase-2 and -9 and their activator kallikrein, in HUVECs [107]. Furthermore, hyperhomocysteinemic patients had elevated mRNA levels of MMP-9 and tissue inhibitors of metalloproteinases-1 (TIMP-1) in freshly isolated peripheral blood mononuclear cells (PBMCs). Most importantly folic acid treatment reduced the levels of homocysteine and concomitantly a significant reduction in the levels of MMP-9 and TIMP-1 mRNA in PBMCs was observed [108].

### Hyperhomocysteinemia: Neurological disorders

**Figure 7:**
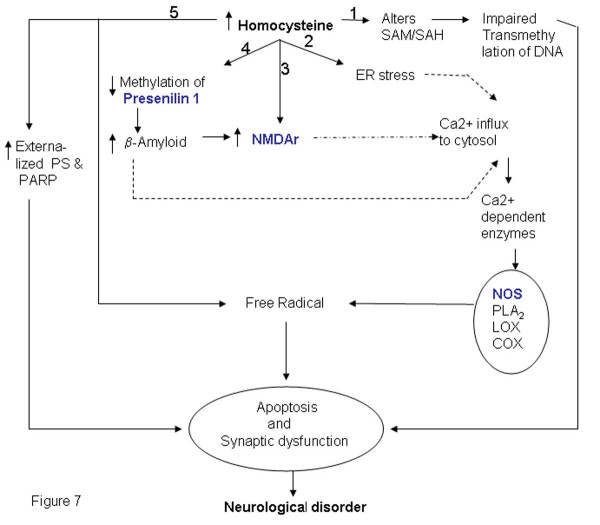
**Pathways that link elevated homocysteine level with Neurological disorders**. Elevated homocysteine levels may cause neurological diseases by various mechanisms which include), altered methylation (branch 1 & 4), ER stress (branch 2), direct interaction with receptors (branch 3) and biomarker of apoptosis (branch 4). The Symbols shaded in color are the gene names given included in the gene-list. The genes marked in blue have been identified by literature based searches as mentioned in the methods section. The solid and dotted lines indicate direct and indirect (multi step) interaction/ conversion respectively.

Elevated levels of homocysteine have been associated with Alzheimer disease (Figure 7). A characteristic feature of the disease is the accumulation of amyloid beta (Aβ) peptide and formation of Amyloid plaques and neurofibrillary tangles in the brain. Although the mechanism of neurodegeneration has not yet been completely elucidated, it is reported that increased calcium levels in the cytosol, increased generation of reactive oxygen species, hyper phosphorylation of tau proteins and apoptosis are the important hallmarks of this disease [109,110]. Increased surface phosphatidyl serine (PS) is an early marker of neuronal apoptosis [111]. The involvement of reactive oxygen species is supported by the fact that high concentrations of copper have been found in the vicinity of Aβ amyloid deposits [112]. Moreover, high levels of antioxidant enzyme such as heme oxygenase-1, SOD-1, catalase and GPX were observed in plaque of brain tissue. Homocysteine also increases the levels of calcium and is known to generate reactive oxygen species especially in the presence of transition metal ions and thus it is not surprising that AD patients show elevated levels of homocysteine. Seshadri et al. found elevated levels of homocysteine prior to disease onset and suggested that the risk of AD increases by about 40% for every 5 μM increase in the concentration of homocysteine [113].

AD patients have elevated levels of homocysteine and decreased levels of SAM. This is believed to alter the DNA methylation status and hence gene expression in AD patients. This hypothesis is supported by the observation that SAM when added to human neuroblastoma SK-N-SH cells in culture, down-regulates expression of PS I gene coding for presenilin, a key factor for Aβ formation in AD due to methylation of its promoter [114]. Similarly, there are other studies suggesting that levels of SAM, folate and vitamin B12 influence DNA methylation of the genes that are involved in the Aβ formation [115]. Furthermore, it has also been reported that Herp, a homocysteine responsive protein, up regulated during ER stress, regulates PS-mediated amyloid beta generation presumably by binding to PS [116].

Homocysteine acts as an agonist and a partial antagonist at the glutamate binding site of the NMDA and the glycine-binding site of the receptor respectively. Under physiological conditions, when the concentration of glycine is normal, the neurotoxicity of homocysteine is observed at a very high concentration (millimolar range). However, under pathological conditions, such as in stroke or trauma where glycine levels in the brain is elevated, neurotoxicity of homocysteine is observed even at very low concentrations of homocysteine (10–100 μM) as the neurotoxic attributes of homocysteine exceeds its protective activity [117]. Under physiological conditions excitation of glutamate receptors initiates the stimulation of lipases and phospholipases with the generation of second messengers that are necessary for normal cell function. However, over stimulation of glutamate receptors leads to excessive calcium entry, abnormal phosphorylation and proteolysis. Thus, increase in the concentration of homocysteine probably results in the over stimulation of glutamate receptors resulting in increased calcium influx. The neuronal damage is due to excess calcium influx and also the accumulation of reactive oxygen species.

Thus, we propose that homocysteine either by inducing oxidative stress or ER stress might lead to apoptosis which in turn may result in neurological disorders. Alternatively homocysteine might act on glutamate receptors triggering a cascade of events that might result in the disease.

## Polymorphisms in genes resulting in hyperhomocysteinemia

Quantitative differences in the activity and availability of enzymes involved in regulation of homocysteine levels directly or indirectly are important in regulating the levels of homocysteine and hence phenotype of complex diseases. The factors that contribute to quantitative variation between individuals are repeat and single nucleotide polymorphism at the genetic level and epigenetic modifications. There are several attempts to analyze polymorphism in genes related to homocysteine pathway. A similar analysis of polymorphism in genes that are part of the interlinked network would be necessary to understand the implications of plasma homocysteine levels on predisposition and manifestation of complex diseases.

Polymorphisms in the genes involved in the methionine and the folate cycles and the transsulfuration pathway (Figure 1) are correlated with elevation of homocysteine levels. A list of genes relevant to homocysteine metabolism along with the known polymorphism and its effect on homocysteine level has been listed (Table 4). Among the genetic polymorphisms MTHFR C677T has been studied extensively. Along with C677T, the 1298A>C and 1793G>A polymorphism were associated with the plasma total homocysteine, folate, and vitamin B12 in kidney transplant recipients [118]. In a recent study we found that in the Indian population MTHFR A1298C but not C677T polymorphism was associated with plasma homocysteine levels [119]. Cystathionine β-synthase (CBS), the first enzyme in transsulfuration pathway, is a B6 – dependent heme protein in mammals. Common mutations in the gene (G919A and T833C) lead to hyperhomocysteinemia, which is directly associated with increased risk of cerebral thrombosis [120]. Furthermore, the most prevalent mutations, a 68 bp insertion in the CBS gene leads to lower increase in post methionine load homocysteine levels while A2756G transition in MS (MTR) gene, are associated with decreased fasting levels of plasma homocysteine [121]. Methionine is converted to SAM by the enzyme methionine-adenosyl transferase (MAT). There are reports suggesting that the expression of MAT is altered in alcoholic liver injury [122]. A tandem repeat polymorphism with the gene coding for thymidylate synthase (TYMS) is known to affect the expression of this enzyme [123]. Reports suggest that TYMS 3/3 genotype is associated with reduced plasma folate and among individuals with low dietary folate intake is associated with elevated plasma homocysteine levels. But the TYMS 3R3R genotype is not a determinant of homocysteine in healthy young Caucasian adults from Northern Ireland [124,125]. There is also an association of a common non-synonymous SNP in the cystathionase (CTH) gene G1346T (S403I) with plasma homocysteine concentrations [126]. Apart from these, glutamate carboxypeptidase II (GCPII) polymorphism (H475Y) is known to elevate the levels of homocysteine/ folate in plasma [127,128].

**Table 4:** An exhaustive list of Gene polymorphism studies that have reported to affects the plasma level of homocysteine

**S.NO**	**Gene Symbol**	**Polymorphism**	**Amino Acid**	**Consequences**	
				**Homocysteine Level**	**Gene expression /Enzyme Activity**

1	MTHFR	C677T A1298C A1793G	A222V E429 A R594Q	↑ ↑ ↑*	↓ [140] ↓ N.R
2	CBS	31 bp VNTR (exon 13-intron 13) G919A 844Ins68 (Exon 8) T833C	----- G307S ----- L278 T	↑**[141–142] ↑ [143–144] ↓ [121] ↑ [120]	↓ ? [120] ↑ (P) [145] ? [120]
3	MTR	A2756G	D919G	↓ [121]	N.R
4	MTRR	A66G	I22M	↑ [145]	N.R
5	MAT	G791A	R264H	NE [146]	↓ [147]
6	TYMS	A 28-bp repeat (Enhancer region) A 6-bp deletion (3'UTR)	-------- -----	↑ [124,125] ↓ (del/del) [148]	Alteration in transcription level[120] ↓ [149]
7	CTH	G1346T	S403I	↑ [126]	N.R
8	GCP II/Folh1	C1561T	H475Y	↑ [127,128]	↓ [128]
9	RFC-1	G80A	R26H	NE [129]	N.R
10	eNOS	G894T T786C (Promoter) CA Repeats (Intron 13)	E298D --------- ---------	↑ [150] ↑ [152] ↑ (In female) [154]	N.E [151] ↓ [153] N.R
11	TC II	C776G A67G	P259R I23V	↑ [130] ↓ [130]	↓ [155]
12	APO E	Epsilon4 alleles	--------	↑ [156]	N.R
13	PAI-1	4G Ins/del (Promoter)	--------	↑ [157]	Affects the response of the PAI-1 promoter to cytokines [158]
14	F2	G20210A (3'UTR)	--------	↑ [159]	↑ [160]
15	Factor V	G1691A	R506Q	↑ [159]	Impairs APC mediated inactivation of factor Va [161]

N.R Not reported in the literature

N .E No Effect was observed.

* Border line association was observed in the presence of high folate concentration.

** After post methionine load

(P) Presence of low concentration of pyridoxal -5-phosphate.

Trascobalamin II (TCN II) facilitates the transport of the vitamin B12 to various tissues. Genetic variations in TCNII gene such as Pro259Arg significantly decrease holo- TCNII or holo-TCNII concentrations [129]. Karin et al have mentioned that cardiovascular disease patients and normal controls, who have high vitamin B12 (>299 pmol/L), tHcy concentrations are lower in individuals homozygous for occurrence of proline259 (259PP) in TCNII protein compared to those with 259PR and 259RR. Therefore, 259PP individuals may be more susceptible to reduction of plasma tHcy in response to increase in vitamin B12 levels [130].

Polymorphism in genes is population dependent. Thus, it might be important to study the status of all these polymorphism in different cohorts to evaluate the importance of each of these polymorphisms with respect to hyperhomocysteinemia.

## Conclusion

The challenges of understanding the molecular etiology of complex diseases is in designing a comprehensive analysis of genetic and epigenetic factors that contribute to quantitative differences in the levels of proteins coded by genes in pathways relevant the disease phenotype. The source of data to derive a rational list of genes for analysis is the literature where interactions and functional relationships between individual gene products have been elucidated. The present study is aimed at generating a resource for selection of genes for polymorphism screening and analysis of mutations as well as epigenetic modification in relation to hyperhomocysteinemia.

We have compiled a gene-list for researchers interested in deciphering the molecular basis of the role of homocysteine as an independent risk factor in cardiovascular diseases and other complex diseases. Among the variety of pathways that are modulated directly or indirectly by the levels of homocysteine, endoplasmic reticulum stress or ER stress emerges as a common pathway affecting different complex diseases. The data compiled here would assist the selection of genes for analysis based on the disease of interest and/or pathways of interest. Presently we are using the gene list for population specific frequency of known SNP and for discovery of new SNP.

It is noted that the levels of Homocysteine may be closely linked to epigenetic effects both as post-replication and post-translation modification. Methylation of histones plays an important role in chromatin remodeling and maintenance of the remodeled state through mitosis. With reference to post-replication modification of CpG sequences homocysteine pathway can function as a auto-regulatory process with reference to methylation of 5'upstream sequences of genes central to its own metabolism: while it can also influence the expression of other genes by regulation of levels of SAM for methylation of 5' upstream sequences. Thus a pathway related analysis of SNP as well as variation at epigenetic level is necessary for complete understanding of the molecular mechanisms relating homocysteine levels and complex disorders.

## List of abbreviations

*B2*: Vitamin B2 ; *B12*: Vitamin B12; *5f-THF*:5-formyltetrahydrofolate; *5,10-CH2-THF*: 5,10-methylenetetrahydrofolate; *5-CH3THF*: 5-methyltetrahydrofolate; *dUMP*: Uridine -5-prime monophosphate; *dUTMP*: Tymidine -5-prime monophosphate; *DMG*: Dimethylglycine; *DMGDH*: Dimethylglycine Dehydrogenase; *M*^+^: Metal catalysed; *ERKP*: Phophorylation of Extracellular signal regulated kinase; *eIF-2α *: Eukaryote initiation factor 2 α ; *CHOP *: C/EBP-homologous protein; *PERK*: PKR-like endoplasmic reticulum eIF2alpha kinase; *CEBPB*: CCAAT/enhancer -binding protein alpha; *EGR1*: Early growth response 1;*CCND1*: Cyclin D1; *IL6R*: Interleukin 6 receptor; *LEP*: Leptin; *LEPR*: Leptin receptor; *PTX3*: Pentraxin 3; *STAT3*: Signal Transducer And Activator Of Transcription 3 *TNFRSF1B*: Tumor Necrosis Factor Receptor Member 1A; *PLA2*: Phospholipase A2; *LOX*: Lipoxygenase; *COX*; Cycloxygenase; *PS*: Phosphatidyl Serine; *PARP*; Poly-ADP-ribosome polymerase
